# Shall Our Horses Wear Shoes?

**Published:** 1881-07

**Authors:** 


### EDITORIAL DEPARTMENT.

### SHALL OUR HORSES WEAR SHOES?

THERE has recently appeared in a leading English monthly an article by Sir George Cox, in which he advocates the plan of letting our horses go unshod. This is, of course, no new idea, but it is now advanced with much specious reasoning by a person whose experience enables him to speak with some authority. The article in question has been very widely circulated and commented upon.

As the question of shoeing or not shoeing is the most vital of all to the health and usefulness of the horse, we propose to review it now at some length. No veterinarian can afford to be without a clear and positive opinion on the matter.

It has been the custom with writers upon the subject of shoeing to class it as a necessary *evil.* We think that this is a mistake. Although the application of shoes is in some instances undoubtedly followed by bad results, yet we believe that the proper adjustment of the horn, and the application of a suitable shoe, is on the whole beneficial, for various reasons.

*First.—Because it prevents the undue expansion of the wall at the plantar surface.*

So much has been said about contraction, that the idea that the hoof may be deformed by expansion may seem improbable, but that such is the case is not an open question. The rearing of an animal of great weight upon moist soil causes the hoofs to assume this form, as witness the feet of most of the Western horses. In these, the wall presents a concave surface externally. This form of wall places the laminal attachments at a disadvantage in sustaining the weight of the animal; the *lateral*

*tension* upon the lamina is excessive; the wall in these flat feet is rendered more susceptible to this lateral pressure by the thinness of the sole.

Such a foot as we have described would be greatly benefited by the application of shoes at an early age. This would prevent the spreading of the hoof, the shortness of the heel, and the thinness of the sole, which will not permit of the use of the animal for one day upon our pavements without suffering from lameness; a fact which is well known to the drivers of such animals.

*Second.—Shoeing is advantageous because it aids in securing and maintaining the foot in its right relation to the limb, and in fitting the animal for the particular work demanded of him.*

Whenever horses are driven for any considerable time without shoes, uneven wear of the hoof is observed. As a rule, this excess of wear is greatest at the outside toe, causing the foot to turn in, a condition indicated by the horseman's phrase "pigeon-toed." These results from the using of horses without shoes have been noted by ancient writers upon the equine foot. In the thirteenth century, a certain traveler, speaking of the Tibetan ponies, which were strong and enduring, and as unlikely to be injured in this manner as any, says, "They are never shod, and the hoof often cracks, and they become pigeon-toed."

Lolleyel, the eminent veterinarian of France, in the seventeenth century, writing of the unshod horses of the German peasantry, says that in nearly every case he found the limbs and feet more or less deformed. These observations are confirmed by many other writers. This uneven wear of the hoof is injurious. It changes the normal bearings of the joints, exciting disease in them. It interferes with the nutrition of the foot. It hinders the development of speed, because the horse requires to be perfectly *balanced* upon his feet to maintain rapid action. For this condition, the application of shoes is an efficient remedy. Iron can be applied to the hoof in such shape as will correct any defect in its form, and when worn can be renewed. The more uneven its wear, the more necessary a frequent renewal.

In reference to the fitting the animal for the work demanded of him: if it is speed that is desired, every trainer knows the efficiency of certain forms of shoes for its development; if for draft, slight calkings vastly increase the animal's power; and if for winter work in countries where much ice is formed, sharp calkings are absolutely indispensable to the horse obliged to pull even a moderate load.

*Third.—Shoeing is a necessity, to prevent destructive wear of the hoof.*

The hoof of the horse, although well enough adapted to withstand the wear incident to the undomesticated condition of the wild horse, is soon worn when forced to travel constantly over our artificial roads. This has been the experience of the world in this matter, and hence the necessity for some protection for the foot, and the iron shoe fastened with nails is the most efficient method yet employed.

The above are, in brief, the principal reasons for advocating the use of shoes.

In the face of these reasons, however, Sir George Cox, after recounting the great variety of shoes in use, and the many evils that come from bad shoeing, characterizes the shoeing of horses as a "wrong-headed and ridiculous system."

He makes this a rule without exceptions. He takes in the whole equestrian field, and makes no allowance for kind of foot-service required or nature of the ground and climate. He gives a few examples of the use of horses without shoes, which he evidently considers sufficient to establish his theory. He seems to have based his conclusions largely upon the experience of past ages, where the circumstances which might assist in determining the question are necessarily difficult to obtain. We select a few of the instances which he gives. The first is of the small, hardy horses of Mexico and South America, that carry packs and make, as he says, "long journeys unshod without injury to their feet." This certainly is no practical test. If the animals carried packs their pace was doubtless a slow one, and opinions might differ as to what constituted an injury. A second instance is the march "of the Cyrean army under Xenophan from the plains of Babylon to the shores of the Euxine," and "from Cunaxa over the

Armenian highlands to the walls of Trebizond; yet we hear nothing of any special difficulties arising from diseases of the foot or leg." These instances are given without the information in regard to the nature of the soil traversed, the season of the year, or service required of the horses, necessary to the forming of a correct estimate of their value. The march might cause the feet to be excessively worn without producing "any special difficulties arising from diseases of the foot or leg."

Another instance which he gives is "the retreat of the French from Moscow." "The horses," he says, "lost all their shoes before they reached Vistula, yet they found their way to France over hard, rough and frozen ground." Of this campaign, history furnishes these facts, viz., that of the 400,000 men of the invading army only 10,000 returned. How many horses "found their way to France?" How they returned to France is thus described by M. Thiers: "Napoleon left on the 6th of November, the army following on the 7th and 8th. The cold had become perceptible, and gave rise to painful regrets at having forgotten to provide winter clothing, and another neglect yet more baffling, that of procuring *frost nails for the shoes of the horses.* Our unfortunate soldiers marched along wrapped up in every kind of clothing saved from the flames of Moscow, and at each ascending portion of the road the artillery horses, even when the usual number required was doubled and trebled, were unable to drag the guns of the smallest calibre. Flogged until they were covered with blood, and their knees torn with frequent falling, they were found incapable of overcoming ordinary obstacles through loss of strength and want of means to prevent slipping on the ice."

Again, the donkeys of Ireland are instanced as animals working successfully without shoes, and the superiority of the horse to this animal in speed and strength is, he thinks, a sure indication that the feet are also superior. He says: "Can one really believe that the animal which is endowed with the greater speed and power should have worse feet than his inferior in both respects?"

This statement needs no refutation, as a very slight acquaintance with the feet of the respective animals will serve to show the superiority of the donkey's. They are thicker in the horn, more concave in the sole, and sustain less weight. Other instances are given of the ability of the foot to withstand wear unprotected by the shoe, but it can be said of them what can truly be stated of those already given, that they are not practical tests.

This theory of Sir George Cox is, as we have said, no new one. Men who, like him, have made the mistake of attributing all the diseases of feet and limbs to shoeing, have also, like him, sought to avoid these evils in a similar manner. But these theories, when subjected to a practical test, have always been found wanting.

It is true that when the animals are small and the feet well formed, with light work in an agricultural district, this method can be employed with tolerable success.

The wear and tear to which we subject our horses to-day is greater than at any previous period. If the smaller and hardier breeds of the ancients were unable to stand this wear without some kind of protection for the feet, how absurd is the idea of subjecting our larger and more delicate-formed animals to such a test over our city pavements in this fast age!

Some of the largest horses in our cities wear out a shoe weighing from four to six pounds in a month. Think of the attrition to which such a hoof would be subject! Even in the country, where feet are well-shaped, continuous use upon the road for a very short time will render them sore in the feet.

No owner of a flat-footed horse need be told of the advantages which shoeing confers. No teamster need be told the difference between the pulling power of the shod and the unshod horse. No experienced horseman but knows the difference between the bold, free, graceful tread of the well-shod horse and the hesitating, "tied-up" gait of the barefooted one. E. A. M.

